# Twelfth scientific biennial meeting of the Australasian Virology Society: AVS12 2024

**DOI:** 10.1128/jvi.02255-24

**Published:** 2025-04-25

**Authors:** Ebony A. Monson, Robson K. Loterio, Justin A. Roby, Anurag Adhikari, Rowena A. Bull, Jill M. Carr, Demetra S. M. Chatzileontiadou, Colin X. Cheng, Fasséli Coulibaly, Samantha K. Davis, Joshua M. Deerain, Mark W. Douglas, Heidi E. Drummer, Nicholas S. Eyre, Wesley Freppel, Anjali Gowripalan, Emma J. Grant, Stephanie Gras, Jenna J. Guthmiller, Lara J. Herrero, Eva Hesping, Bethany A. Horsburgh, Jennifer L. Hyde, Marios Koutsakos, Jason M. Mackenzie, Jackie E. Mahar, Laura C. McCoullough, Christopher L. D. McMillan, Naphak Modhiran, Rhys H. Parry, Damian F. J. Purcell, Daniel J. Rawle, Andrii Slonchak, Peter G. Speck, Gilda Tachedjian, Thomas Tu, Gregory W. Moseley, Johanna E. Fraser, Michelle D. Tate

**Affiliations:** 1 Department of Microbiology, Anatomy, Physiology and Pharmacology, La Trobe University 422090, Bundoora, Victoria, Australia; 2 Infection and Immunity Program, La Trobe Institute for Molecular Science (LIMS), La Trobe University 2080, Bundoora, Victoria, Australia; 3 Life Sciences Discipline, Burnet Institute 104125, Melbourne, Victoria, Australia; 4 Department of Microbiology, Biomedicine Discovery Institute, Monash University 214149, Clayton, Victoria, Australia; 5 Training Hub promoting Regional Industry and Innovation in Virology and Epidemiology (THRIIVE), Gulbali Institute, Charles Sturt University 1109, Wagga Wagga, New South Wales, Australia; 6 Department of Biochemistry and Chemistry, School of Agriculture, Biomedicine and Environment (SABE), La Trobe University 264997, Bundoora, Victoria, Australia; 7 Department of Infection and Immunology, Kathmandu Research Institute for Biological Sciences, Lalitpur, Nepal; 8 School of Biomedical Sciences, Faculty of Medicine, University of New South Wales Sydney 7800, Sydney, New South Wales, Australia; 9 Kirby Institute, University of New South Wales Sydney 7800, Sydney, New South Wales, Australia; 10 College of Medicine and Public Health, Flinders Health and Medical Research Institute, Flinders University 198094 , Adelaide, South Australia, Australia; 11 Department of Biochemistry and Molecular Biology, Monash University 198101 , Clayton, Victoria, Australia; 12 Infection Program, Biomedicine Discovery Institute, Monash University 161661, Clayton, Victoria, Australia; 13 Centre for Disease Preparedness, Commonwealth Scientific and Industrial Research Organisation (CSIRO), Geelong, Victoria, Australia; 14 Storr Liver Centre, The Westmead Institute for Medical Research, The University of Sydney at Westmead Hospital, Westmead, New South Wales, Australia; 15 Centre for Infectious Diseases and Microbiology, Sydney Infectious Diseases Institute, The University of Sydney at Westmead Hospital, Westmead, New South Wales, Australia; 16 Department of Microbiology and Immunology, The Peter Doherty Institute for Infection and Immunity, University of Melbourne 198084, Parkville, Victoria, Australia; 17 Institute for Biomedicine and Glycomics, Griffith University 5723, Southport, Queensland, Australia; 18 John Curtin School of Medical Research, Australian National University 10110 , Canberra, Australia; 19 Department of Immunology and Microbiology, University of Colorado Anschutz Medical Campus 129263, Aurora, Colorado, USA; 20 Department of Microbiology, University of Washington 312771, Seattle, Washington, USA; 21 Victorian Infectious Diseases Reference Laboratory, Royal Melbourne Hospital at The Peter Doherty Institute for Infection and Immunity 90134, Melbourne, Victoria, Australia; 22 Department of Microbiology and Immunology, University of Melbourne at the Peter Doherty Institute for Infection and Immunity, Melbourne, Victoria, Australia; 23 School of Chemistry and Molecular Biosciences, The University of Queensland 198110 , Saint Lucia, Queensland, Australia; 24 Australian Infectious Diseases Research Centre, Global Virus Network Centre of Excellence, Brisbane, Queensland, Australia; 25 Infection and Inflammation Program, QIMR Berghofer Medical Research Institute 56362, Herston, Queensland, Australia; 26 College of Science and Engineering, Flinders University 117627, Bedford Park, South Australia, Australia; 27 Centre for Innate Immunity and Infectious Diseases, Hudson Institute of Medical Research 366840 , Clayton, Victoria, Australia; 28 Department of Molecular and Translational Sciences, School of Clinical Sciences, Monash University 2541, Melbourne, Victoria, Australia; The University of Arizona, Tucson, Arizona, USA

**Keywords:** innate immunity, virus-host interactions, animal viruses, antivirals, bacteriophages, clinical virology, epidemiology, immunology, vaccines

## Abstract

The Australasian Virology Society (AVS) holds premier biennial virology meetings that foster multidisciplinary research and collaboration and promote equity and inclusion of early-career researchers. The 12th AVS meeting (AVS12), convened by M. Tate, J. Fraser, and G. Moseley, was held from 2 to 5 December 2024 on Dja Dja Wurrung country at the RACV Goldfields Resort in Creswick, Victoria, Australia. In this report, we give a brief overview of the history of AVS and outline the current and developing priorities for the society. We provide a summary of the insightful panel discussions held to address career development and Indigenous virology, highlight the presentations given by international plenary speakers Joe Grove and Chantal Abergel, and celebrate the recipients of the numerous awards.

## INTRODUCTION

The Australasian Virology Society (AVS) biennial meeting is highly recognized as the premier virology conference in Australasia, fostering collaboration across diverse fields of virology and promoting multidisciplinary research. It provides an engaging platform for researchers, including early-career researchers (ECRs), to share their findings on human, animal, prokaryotic, and plant viruses. The 12th AVS meeting (AVS12) was held from 2 to 5 December 2024 on Dja Dja Wurrung country, at the RACV Goldfields Resort (Creswick, Victoria, Australia). AVS12 was attended by 280 delegates—a record number for this conference—demonstrating the ongoing growth and success of the virology community in Australasia. This report provides an overview of the history of the AVS, highlights the current focus of the society, and provides insights into ECR and Indigenous virology initiatives.

## HISTORY OF AVS

The inaugural AVS meeting, originally called the Australian Virology Group (AVG), was founded by Paul Young in 2001 to provide a dedicated scientific meeting for virologists in Australia. The concept was embraced by the virology community with the first scientific meeting held at the Kingfisher Bay Resort on K’gari (Fraser Island, Queensland, Australia). The meeting had two major objectives. The first was to provide an enjoyable and productive forum for researchers to present and discuss their work among their collaborators and other virology researchers. The second was to provide a venue to foster and encourage the participation of our students and ECRs, whereby registration costs were kept to a minimum and sponsorship was sought to provide travel scholarships. The first meeting was held over 5 days in December 2001, with 230 delegates attending, approximately one-third of whom were undergraduate and PhD students. AVG was a relatively informal grouping, with no official structure and governance and continued to host biennial meetings. However, after nearly 10 years, the option was raised of formalizing the group as a society so that it could fulfill a wider objective as an advocacy and lobby group for our discipline. The AVG was incorporated into the AVS in 2010, formalizing the organization as a legal entity with an appropriate governance structure and constitution. Paul Young served as the first president of AVS until 2011, laying the foundation for its growth and success (1).

**Fig 1:**
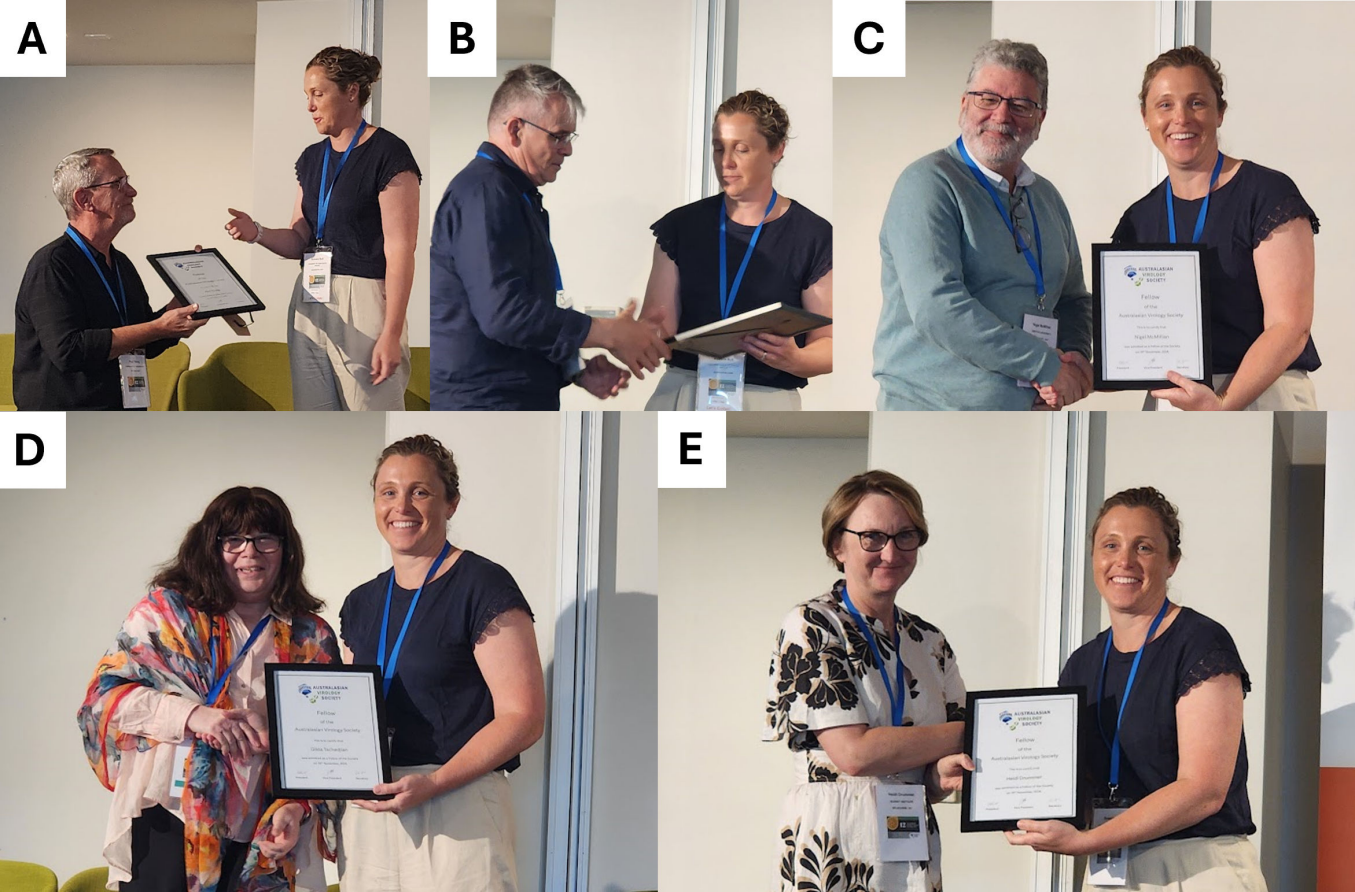
AVS past presidents receiving their FAVS, presented by the current president, Rowena Bull. (**A**) Paul Young, (**B**) Damian Purcell, (**C**) Nigel McMillan, (**D**) Gilda Tachedjian, and (**E**) Heidi Drummer.

From the fledgling AVG, the AVS has evolved into a mature society with gender equity and diversity underpinning the society’s vision and activities, under the successive distinguished leadership of Damian Purcell (2011–2015), Nigel McMillan (2015–2017), Gilda Tachedjian (2017–2021), and Heidi Drummer (2021–2023). In recognition of their exceptional contributions to advancing AVS and fostering excellence in the field of virology, at AVS12, they were presented with the inaugural “Fellowship of the AVS (FAVS)” by the current president, Rowena Bull (2023–present) (Fig. 1).

## AVS TODAY

AVS has evolved into a highly productive organization, with over 260 current members spanning eight countries, which promotes, supports, and advocates for the discipline of virology. The Australasian region harbors a diverse and unique virome, shaped by its geographical isolation and distinctive flora and fauna. One of the society’s foundational goals is mentoring the next generation of virologists to maintain strong expertise in the field throughout the Australasian region. This commitment is evident in our annual meetings, where early-career members frequently present and hold chair positions.

As global warming accelerates and the world becomes increasingly interconnected, viruses continue to pose significant catastrophic potential. While AVS traditionally focused on virologists in Australia and Aotearoa (New Zealand), the society has recently expanded its reach through online seminars and engagement with other nations in the Australasian region and beyond—a natural progression given our shared virological challenges. We also established a reciprocal program with the American Society of Virology (ASV), sending emerging leaders in virology to each other’s meetings to foster collaborative research. As our society matures, we have become increasingly mindful of how virological research in Australia impacts Indigenous communities. We are currently drafting recommendations for virologists conducting research on Indigenous lands.

AVS recognizes the challenging times facing our field. In the wake of the COVID-19 pandemic, we have observed increasing vaccine hesitancy, reflected in the resurgence of highly contagious viruses like measles. In response, AVS is collaborating with our members to create engaging educational videos that address relevant public health issues for the broader community.

## OVERVIEW OF AVS12

In 2024, AVS12 featured two distinguished international keynote speakers, 12 additional plenary speakers, 46 presentations selected from submitted abstracts, 15 rapid-fire talks by ECRs, and over 160 poster presentations. The conference encompassed a comprehensive and insightful program across a broad spectrum of virology and related disciplines, with sessions covering innate and adaptive immunity, virus–host interactions, animal viruses, antivirals, bacteriophages, clinical virology, epidemiology, Indigenous virology, structural virology, and vaccines. Several initiatives were introduced at AVS12, including a new ECR career development session, as well as a panel which discussed how the society could promote excellence in Indigenous virology.

The conference began with a formal Welcome to Country delivered by Djaara Traditional Owner Mr. Lewis Brown. It was an honor to be Welcomed, to hear of Djaara people’s deep connection to their land, and to appreciate the intersection between language, culture, and knowledge that is understood by this country’s first scientists. AVS strongly advocates for the respectful acknowledgment of, and meaningful engagement with, the Indigenous peoples of our region, which was further discussed during the Indigenous virology panel session.

**Fig 2:**
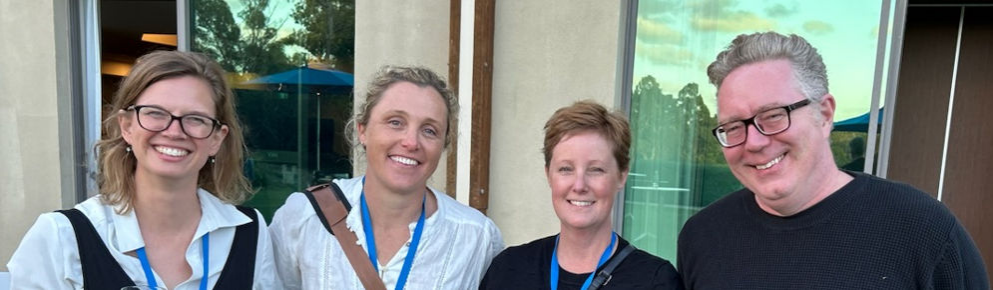
AVS12 Conveners with the current AVS President. L-R: Johanna Fraser (Convenor), Rowena Bull (AVS President), Michelle Tate (Convenor), and Gregory Moseley (Convenor).

The AVS12 Local Organizing Committee (LOC) (Table 1) delivered a highly successful meeting led by Meeting Convenors Johanna Fraser, Michelle Tate, and Gregory Moseley (Fig. 2). AVS12 would not have been possible without the generous support from our sponsors and exhibitors (see Acknowledgments).

**TABLE 1:** AVS12 LOC

Committee member	Committee role
Johanna Fraser	Meeting Convener/budget/venue/Indigenous representation, diversity, inclusion
Gregory Moseley	Meeting Convener/Indigenous representation, diversity, inclusion
Michelle Tate	Meeting Convener/budget/sponsorship/Indigenous representation, diversity, inclusion
Rowena Bull	AVS President
Jason Mackenzie	AVS Vice-President/program/Indigenous representation, diversity, inclusion
Peter Speck	Program/Indigenous representation, diversity, inclusion
Mark Douglas	Program
Peter White	Sponsorship
Karla Helbig	Networking events
Sarah Londrigan	Networking events
Kathryn Edenborough	Awards and prizes
Stacey Lynch	Awards and prizes
Jody Hobson-Peters	Abstracts
Fasséli Coulibaly	Abstracts/Indigenous representation, diversity, inclusion
Ebony Monson	ECR sub-committee
Robson Kriiger Loterio	ECR sub-committee
Gabriela Khoury	ECR sub-committee
Justin Roby	Indigenous representation, diversity, inclusion
Robin MacDiarmid	Indigenous representation, diversity, inclusion
Heidi Drummer	*Ex officio* (AVS President)
Gilda Tachedjian	*Ex officio* (AVS President and ACH4 representative)

### Equity and diversity at AVS12

The AVS has a strong commitment to promoting inclusiveness, equity, and the prevention of discrimination (2). This includes ensuring appropriate inclusion of different ethnicities, genders, career stages, and researchers from different states and institutions across Australasia. At AVS12, there were several initiatives that supported our policy on equity and diversity, including a travel grant for an Indigenous delegate to attend AVS12 and 4 travel grants for delegates from underrepresented states/regions (outlined below and in Table 2).

**TABLE 2:** AVS12 travel grants

Award	Awardee	Presentation title
Indigenous Delegate Travel Grant	Natalie Netzler	Harnessing the antiviral activities of Pacific traditional medicines
Scientists from Underrepresented State/Regions Travel Grant	Babu Nath	High-resolution structure of the circovirus replication associated protein
	Natalie Watson	Antiviral potential of PI3K inhibitors against herpes simplex virus
	Anjali Growripalan	Using oncolytic viruses to save Tasmanian devils from transmissible facial tumours
	Wesley Freppel	Inhibitor A11N targets the protease region of alphavirus capsid, inhibiting early viral replication, and demonstrates broad-spectrum antiviral activity against flaviviruses
Australian Centre for Hepatitis Virology (ACHV) Travel Grant	Harout Ajoyan	Evidence of chromosomal translocation, complex integration patterns, and pre-genomic RNA expression in *de novo* hepatitis B virus integration
	Laura McCoullough	Using CRISPR-Cas13b as a novel antiviral to target the hepatitis B RNAs to suppress hepatitis B virus replication and protein expression *in vitro* and *in vivo*

**Fig 3:**
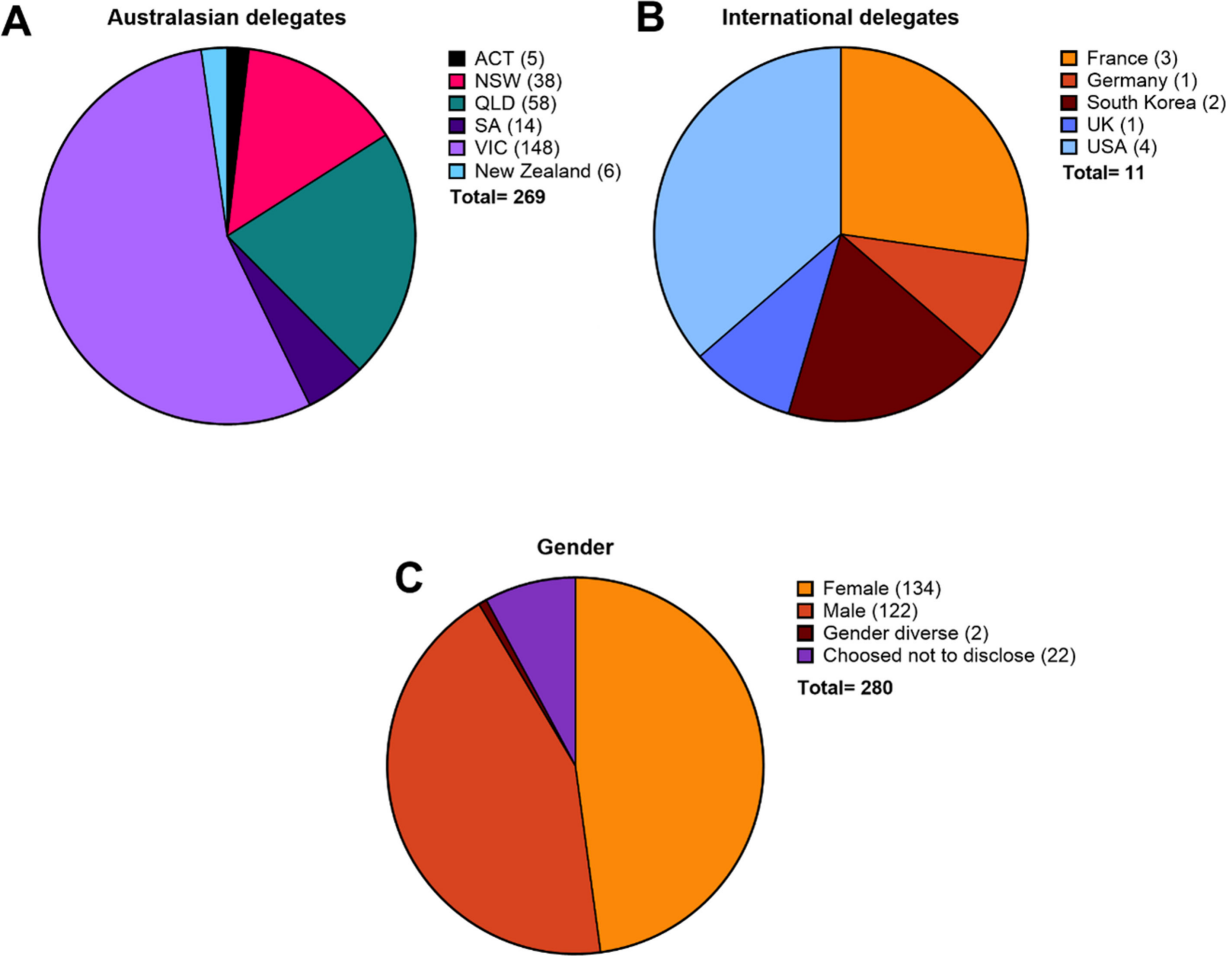
Breakdown of attendees at AVS12. (**A**) Breakdown of Australasian delegates and (**B**) International delegates. (**C**) Gender breakdown of attending delegates.

There were a record-breaking 280 delegates at AVS12, with 269 delegates from Australasia: Victoria (53%), followed by Queensland (21%), New South Wales (14%), South Australia (5%), Australian Capital Territory (2%), and New Zealand (2%) (Fig. 3A). Eleven delegates attended from the United States, France, South Korea, Germany, and the United Kingdom (6%; Fig. 3B). Notably, approximately 57% of registrants were ECRs. In addition, approximately 50% of attendees identified as female (Fig. 3C). This strong participation underscores the conference’s efforts toward fostering an inclusive environment. To support and encourage parents to attend and engage with the meeting, a carers room was available which included activities for children and a large screen which live streamed all presentations.

## KEY PLENARY PRESENTATIONS

### Ruth Bishop oration

**Fig 4:**
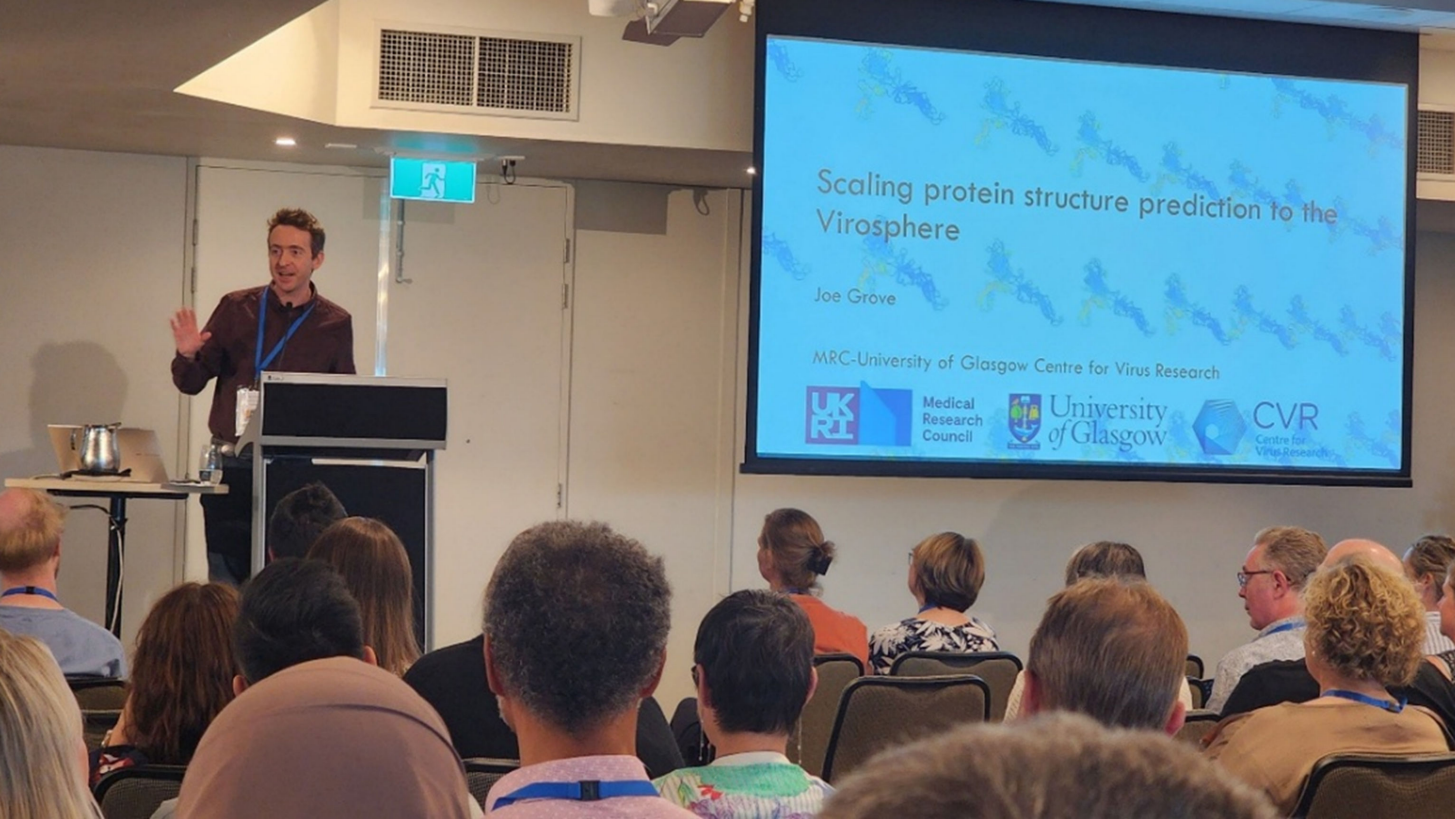
Joe Grove presenting the Ruth Bishop Oration.

The Ruth Bishop oration was provided by Joe Grove (The University of Glasgow, Scotland) on “Scaling protein structure prediction to the virosphere” (Fig. 4). Joe presented an engaging opening talk describing how significant advances in the use of artificial intelligence to predict protein structures can be applied to study the evolution of viral glycoproteins as applied to the *Flaviviridae*. While most of the glycoproteins of the orthoflaviruses, including divergent jingmenviruses and large genome flaviviruses, display a typical class II fusion protein fold, the E1 and E2 glycoproteins of the hepaciviruses, pegiviruses, and pestiviruses have a unique fold and may represent a new class of fusion proteins. Comparison of cryo-electron microscopy (cryo-EM) structures of intact and chimeric E1/E2 glycoproteins closely resembled the predicted structures of E1/E2. The use of structural prediction programs such as AlphaFold and ESMFold provides a new tool with which to identify the location within the genome of potential viral glycoproteins, the evolution of viral glycoproteins, and new classes of fusion proteins. The work was published in *Nature* (3).

### Rob Webster oration

**Fig 5:**
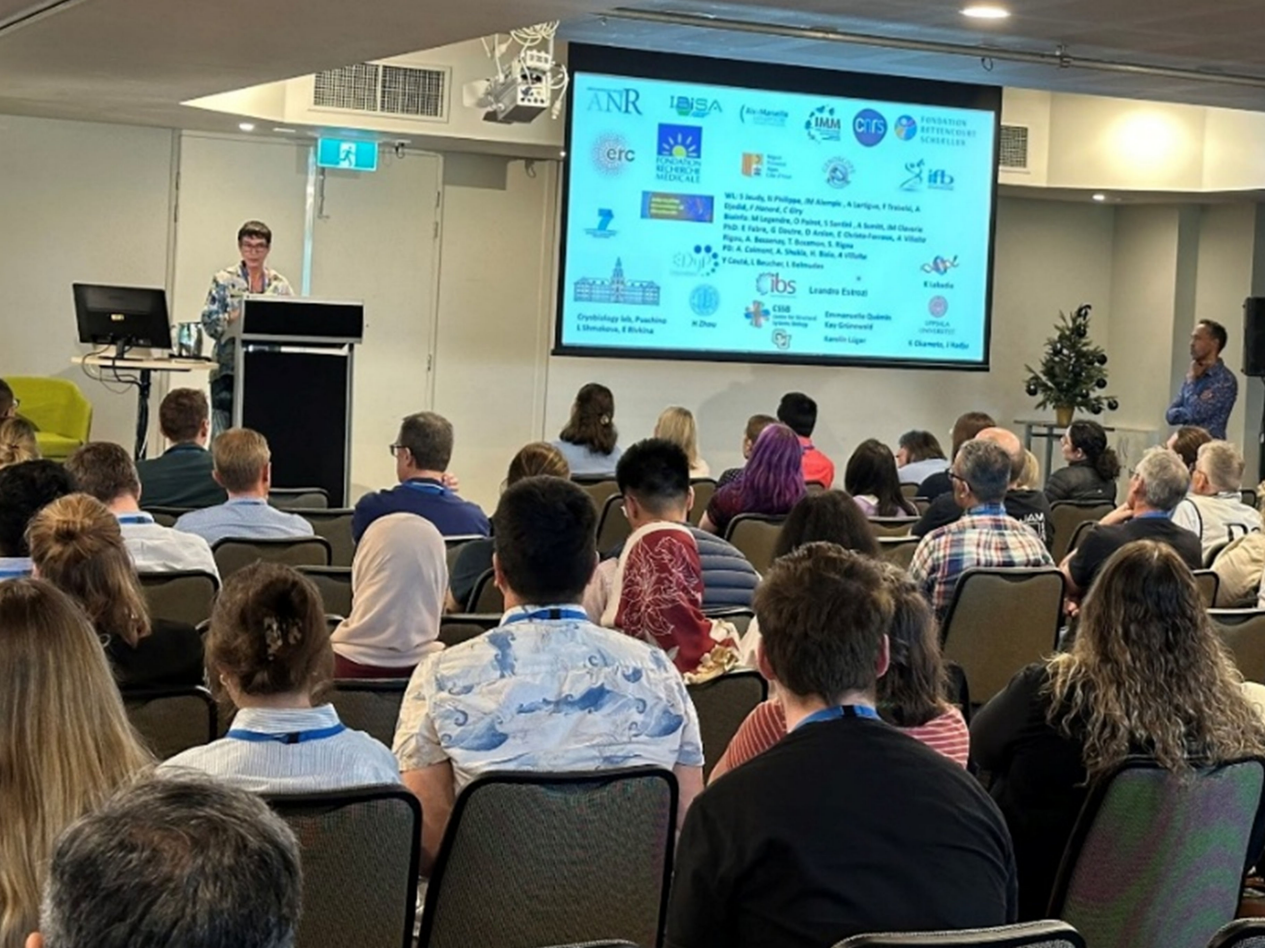
Chantal Abergel presenting the Rob Webster Oration.

In keeping with the oration’s namesake, Chantal Abergel (CNRS and Aix Marseille University, France) presented a vast virus discovery program spanning over 20 years (4) and reaching across the globe (Fig. 5). This program led to the identification of novel viruses characterized by their unprecedented size and complexity. These findings quickly overturned the initial view that giant viruses are an anomaly in the virosphere. Instead, they were found to be ubiquitous and diverse, lurking in 30,000-year-old permafrost (5), as well as in plain sight in our own backyard (6). These viruses are visible by light microscopy and boast up to 2,500 genes, more than some eukaryotic organisms. Beyond the discovery phase, Chantal described how the field has now moved toward a functional understanding of the unique biology of giant viruses thanks to robust reverse genetic systems and powerful structural biology approaches. These insights led to the identification of several hallmarks, such as membrane-less organelles called viral factories, which coordinate viral replication and assembly (7). Despite their large size, some of these viruses also evolved various strategies to compact and package the genome using virally encoded histones for Melbournevirus (8) or helical fiber-forming proteins for Mimivirus (9). Many of these viruses even have their own virophages, smaller viruses that may “infect” their associated giant virus or remain commensal (10). These two decades of bold research have mapped what used to be *terra incognita* within the virosphere. In doing so, they have unearthed a trove of unique particle architectures, biosynthetic pathways, and protein machinery that promise to be an exciting area of research for the years to come.

### Paul Young plenary

**Fig 6:**
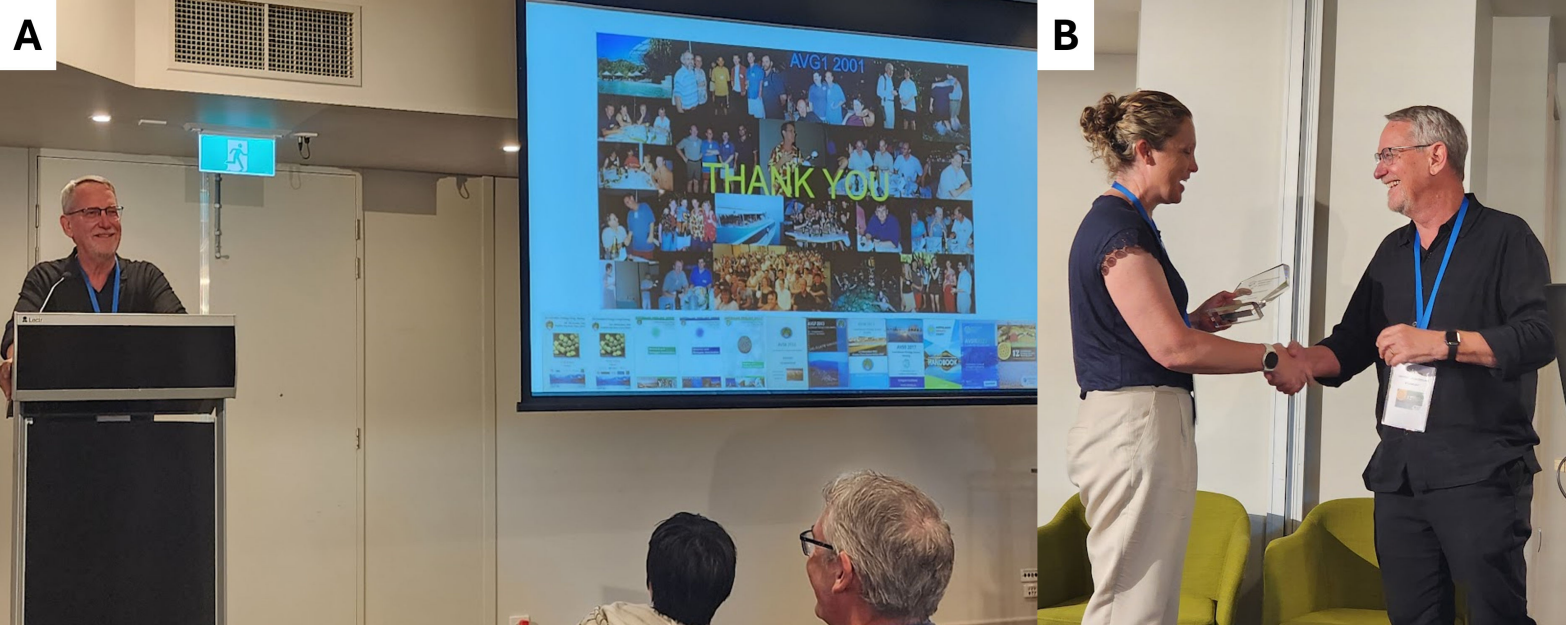
Paul Young Plenary. (**A**) Paul Young delivering a plenary “A Virologist’s Adventures in Wonderland.” (**B**) Paul was honored with a life membership of AVS, presented by current president, Rowena Bull.

In their plenary, Paul Young (University of Queensland), the founder of AVS, detailed the society’s history and development into a pivotal advocacy group for virology in Australasia (Fig. 6A). Paul recounted personal anecdotes and significant milestones, emphasizing the society’s evolution and highlighting the importance of community and shared knowledge. He also provided some reflections on his journey, with advice to both young scientists and his peers. Finally, Paul pointed to the future and the power of partnerships between academia, industry, and government, highlighting the role the society could, and should play in helping drive a strategic national agenda for transforming the sector. In recognition of his leadership, Paul was honored with an AVS life membership (Fig. 6B).

## HIGHLIGHTING INDIGENOUS VIROLOGY AT AVS12

AVS recognizes that Indigenous communities maintain thousands of years of accumulated knowledge regarding traditional medicines and observations on the patterns of disease transmission and resolution. Since 2019, AVS has held an Indigenous virology session at our biennial meeting (1), aiming to highlight the important research undertaken by Indigenous scientists and research concerning viruses that disproportionately affect Indigenous communities. At AVS12, we aimed to reassess how we can better support Indigenous researchers; enhance Indigenous engagement in science, technology, engineering, and mathematics; and respectfully engage with Indigenous communities and lands in our research.

**Fig 7:**
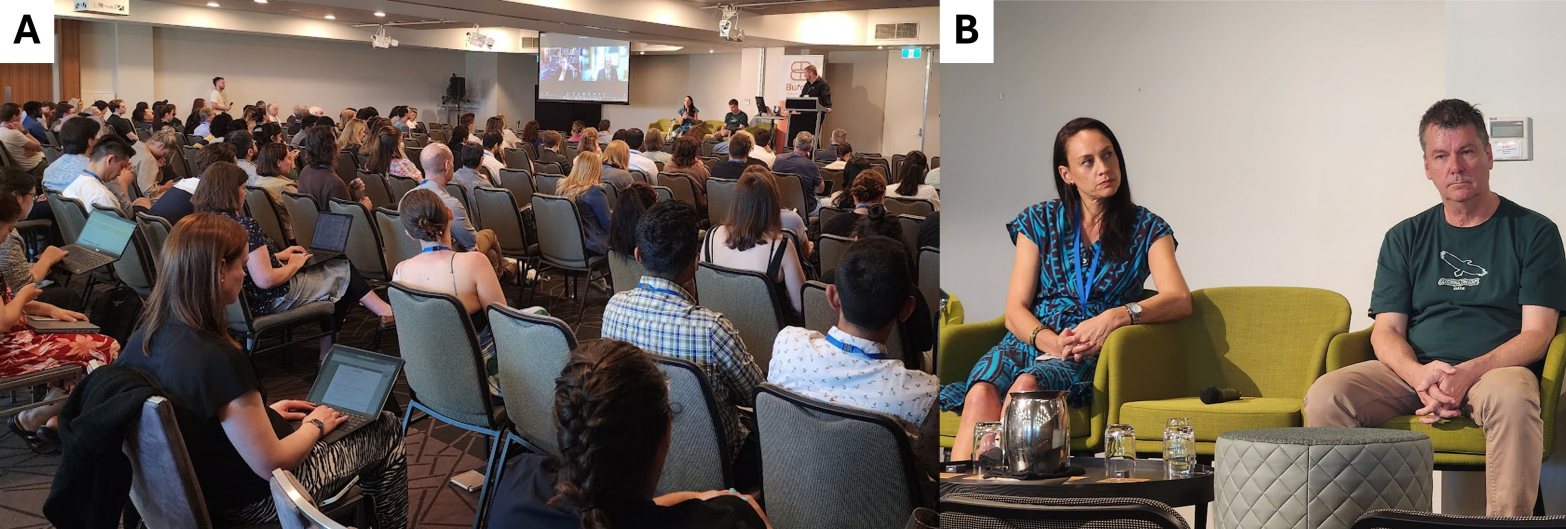
Indigenous virology session at AVS12. (**A**) Indigenous virology panel discussion, with panel members (**B**) Natalie Netzler (left) and Trevor Lithgow (right). Lloyd Dolan and Allison Imrie participated virtually.

To do this, AVS12 introduced a 1-hour panel discussion held during the Indigenous virology session. The panel, eloquently chaired by Justin Roby (Charles Sturt University), focused on “What can we do as a society to better promote Indigenous representation and excellence in Virology?”. The discussion aimed to identify barriers to Indigenous student engagement and propose initial strategies to bridge this gap, fostering a positive legacy in the field. The panel featured Natalie Netzler (University of Auckland, New Zealand) of Sāmoan (Moto’otua, Falealili) and Māori (Ngāti Ruanui, Ngāti Hauā) heritage; Trevor Lithgow (Monash University)—who has worked with Indigenous communities to pioneer approaches for ethical research; Lloyd Dolan (Charles Sturt University) of Wiradjuri heritage; and Allison Imrie (University of Western Australia) of Indigenous Tongan heritage, who brought diverse Indigenous perspectives (Fig. 7). Topics included pathways to support Indigenous virology students, reflections on Indigenous experiences in the workplace, the use of Indigenous species in our research, and the integration of Indigenous knowledge into modern science. The panel concluded that scientific progress benefits from local environmental and contextual interactions, emphasizing the need for greater involvement of and partnership with Traditional Owners and Custodians in future research endeavors.

Immediately following the panel discussion, Trevor Lithgow presented his research ventures into ethical bioprospecting for bacteriophages. Working collaboratively with the Wurundjeri Woi Wurrung Cultural Heritage Aboriginal Corporation, the Lithgow laboratory was able to leverage traditional knowledge and prospect for bacteriophages isolated within the Merri Creek (Melbourne, Australia). This led to the discovery of novel, minimalist phages that infect and inhibit the growth of clinical isolates of *Klebsiella pneumoniae* (11). Their naming by Wurundjeri elders as Merri-merri-uth nyilam marra-natj (phage MMNM; meaning “dangerous Merri lurker” in Woi wurrung language) was presented with a characterization of their activity. The minimalist architecture of the phage is original and particularly attractive for understanding and manipulating virus–host interactions. Natasha Jansz (Doherty Institute) then presented their research on human T-cell lymphotropic virus type 1c (HTLV-1c), a retrovirus that is endemic in Central Australian First Nations communities, with a prevalence of up to 40%. Infection with HTLV-1c leads to an increased risk of chronic lung disease and early death. Natasha’s humanized mouse model coupled with long-read sequencing allowed in-depth analyses of integrated HTLV-1 genome structure and integration site selection.

## SUPPORTING AND PROMOTING EARLY AND MID-CAREER VIROLOGY RESEARCHERS AT AVS12

A key aspect of the AVS mission is to promote, encourage, support, and foster the development of early career researchers (ECRs; ≤10 years post-PhD), as well as mid-career researchers (≤15 years post-PhD), by providing them with valuable opportunities to present their work and engage with the broader virology community. This commitment is evident through the inclusion of ECRs throughout the program of biennial meetings and virtual symposia, where ECRs can showcase their research and participate in panel and career development discussions, chair sessions, and network with established professionals.

### Career development sessions

**Fig 8:**
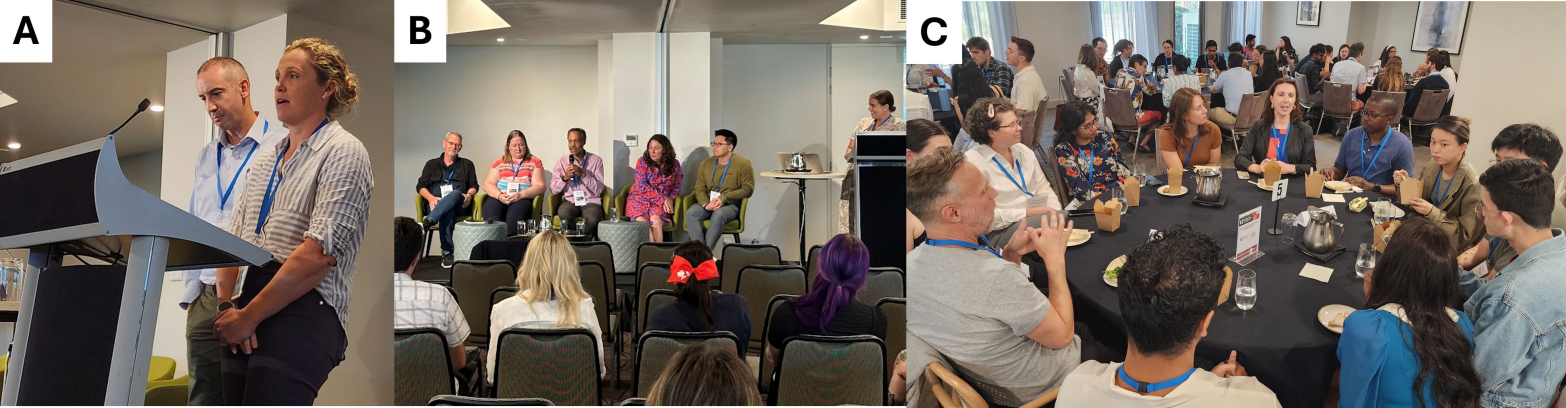
Career development sessions at AVS12. (**A**) Rowena Bull and David Williams delivering the first session, “grant writing and networking.” (**B**) Panel discussion during the career development forum. (**C**) Discussions at the “Meet the Professor’s Lunch.”

Three career development sessions were held at AVS12 that were very well attended. Unlike previous AVS meetings, two sessions were strategically scheduled before the official conference opening, thereby setting the stage and providing ECRs with additional time to apply the knowledge acquired throughout the conference. Moderated by Ebony Monson (La Trobe University) and Robson Loterio (Burnet Institute), the first session was attended by over 100 ECRs. Presentations by Rowena Bull (University of New South Wales) and David Williams (Commonwealth Scientific and Industrial Research Organisation [CSIRO]) (Fig. 8A) focused on grant writing and networking, emphasizing the critical importance of professional networks in the scientific community. This was followed by a panel discussion featuring prominent researchers, Paul Young (University of Queensland), Stephanie Gras (La Trobe University), Fasséli Coulibaly (Monash University), Stacey Lynch (CSIRO), and Byron Shue (National Institute of Health, USA) (Fig. 8B). The panelists discussed various topics, such as navigating career transitions, securing funding, and building collaborative research projects.

The “Meet the Professor’s Lunch” brought together over 120 ECRs, providing them with a unique opportunity to engage with 22 Professors or Associate Professors, informally and directly (Fig. 8C). The event facilitated meaningful exchanges, inspired new ideas, and strengthened the sense of community.

### AVS12 travel grants

AVS is committed to enabling the next generation of virology researchers to attend our meetings. AVS12 offered seven travel grants (Table 2) that were generously supported by our sponsors (see Acknowledgements). Of these travel grants, four delegates from underrepresented states/regions outside of Australia’s four major cities were sponsored to attend. Two viral hepatitis researchers were also awarded travel grants sponsored by the Australian Centre for Hepatitis Virology (ACHV). Lastly, a new initiative for AVS12 was a travel grant that aimed to support the attendance of a delegate who identifies as Indigenous. Collectively, these travel grants promoted diversity at AVS12 and provided emerging leaders the opportunity to present their findings and foster new collaborations.

Another new initiative of AVS12 was the AVS-ASV exchange program, which intends to highlight the outstanding virology research from the United States and Australasia at the AVS and ASV meetings, respectively. The ASV and AVS sponsored the travel of the outstanding ECR Jenna Guthmiller (University of Colorado, USA), the recipient of the 2024 ASV Ann Palmenberg Junior Investigator Award. Jenna presented their advances in understanding the role of antibodies in driving antigenic drift of influenza A viruses.

### AVS12 awards

In addition to the travel grants, 15 awards were presented to ECRs at AVS12. Table 3 outlines the award winners. These awards were made possible through generous support from our sponsors (see Acknowledgments). Five ECRs were recognized with prestigious awards for high-quality oral presentations. One of these awards included registration and travel costs to attend the World Society for Virology (WSV) conference in Kuala Lumpur in May 2025. AVS12 also provided the opportunity for ECRs to present their novel research findings during two sessions of 3-minute oral presentations. The audience was asked to vote on the spot, and the top 2 presenters were selected to receive the ”People’s Choice” awards. In addition to the main program, two poster sessions were held, providing endless opportunities for networking and gaining constructive feedback. Four outstanding poster presenters were recognized with awards that were kindly sponsored by the American Society for Microbiology, Journal of Virology. Lastly, an industry engagement award, which was selected based on the top-ranked eligible abstract, was a new initiative introduced at AVS12 that acknowledged and recognized excellence in industry-engaged virology research.

**TABLE 3:** AVS12 awards

Award	Awardee	Presentation title
Emerging Leadership		
AVS12 Rising Star	Natalee Newton	Chimeric viral platform enables high-resolution cryo-EM analysis and antigenic characterization of diverse pathogenic tick-borne flaviviruses
The Young Award	Lara Herrero	Rethinking the way we treat viral-induced diseases: beyond the use of antivirals
Industry Engagement Award	Caolingzhi Tang	Polyphenol rich sugarcane extract (PRSE) has antiviral activity against influenza A virus *in vitro*
Research Presentations		
Science Bites People’s Choice Award	Jacinta Agius	Protecting Australia’s abalone: A herpesvirus (HaHV-1) defence strategy
	Ellesandra Noye	Natural killer cells in children with obesity have a “trained” immune phenotype and heightened pro-inflammatory responses to *ex vivo* influenza A virus stimulation
Poster Presentation Awards	Ryan Johnston	Integrating a chimeric Binjari virus nanotechnology into paper-based assays for POC detection of flaviviral infections in veterinary applications
	Adam Lopez-Denman	REAPER: Mosquito *in vivo* virus targeting to control viral transmission
	Henry Munyanduki	Investigation into the ability of Victorian hematophagous insects to acquire and retain lumpy skin disease virus
	Connor Scott	Structural characterisation of anti-JEV human antibody epitopes reveals a new target for broad spectrum therapies and vaccines
ECR Oral Presentation Awards	Mary Petrone	Extending the evolutionary history of disease-causing RNA viruses
	Angela Harrison	Dissecting viral protein multifunctionality: a path to understanding how deadly RNA viruses remodel the host cell
	Christopher McMillan	Using spatial transcriptomics to uncover the immune mechanisms of microarray patch vaccination
	Bethany Horsburgh	A novel long-range sequencing assay for HIV and HCV for use in a diagnostic setting
	Ariel Isaacs	A protective bispecific antibody targets both Nipah virus surface glycoproteins and limits viral escape
World Society for Virology (WSV) Award	Anjali Gowripalan	Using oncolytic viruses to save Tasmanian devils from transmissible facial tumours

**Fig 9:**
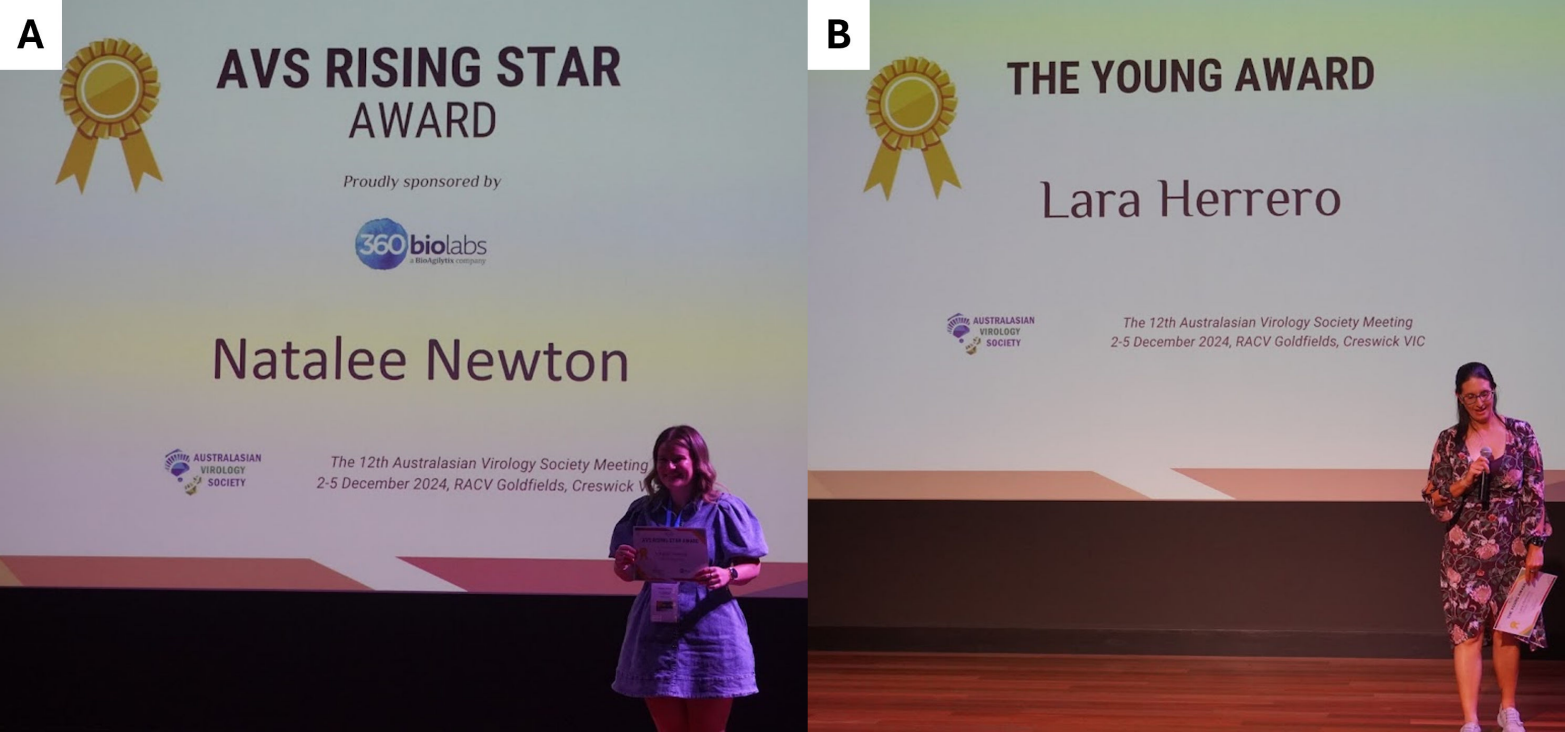
Prestigious AVS12 awards. (**A**) Natalee Newton receiving the AVS Rising Star Award. (**B**) Lara Herrero receiving the inaugural AVS Young Award.

The winner of our most prestigious AVS prize, the “AVS Rising Star Award” for an ECR ≤5 years post-PhD demonstrating potential as a future leader in the virology discipline, was won by Natalee Newton (University of Queensland) (Fig. 9A). This award was selected based on the quality of the oral presentation, contributions to the field, and evidence of emerging leadership. Newton delivered an outstanding presentation on their work on high-resolution cryo-EM analysis and antigenic characterization of diverse pathogenic tick-borne flaviviruses.

Finally, AVS12 presented a new award, “The Young Award.” This award honors the legacy of the AVS founder Paul Young and their outstanding contributions to virology and the effort they put into supporting and developing our society for more than two decades. The award recognizes an outstanding mid-career researcher who has made substantial contributions to AVS and the discipline of virology, either within or beyond the scientific community. Lara Herrero (Griffith University) was the recipient of the inaugural AVS Young Award (Fig. 9B) for significant contributions to the development of antivirals for the treatment of alphavirus-induced arthritis. Professor Herrero has contributed significantly to the AVS, convening the 11th AVS meeting (AV11; Gold Coast, Australia) and serving as an AVS committee member for several years.

## FINAL REFLECTIONS

Overall, the AVS12 meeting showcased the breadth and quality of virology being undertaken in the Australasian region, spanning discovery science, translational, clinical, and epidemiological studies. The AVS consistently promotes the talent of our budding virologists and focuses on equity and diversity in our research and within our meetings. AVS12 promoted ECR career development and Indigenous virology and hopes to take the learnings from our first Indigenous virology panel session forward and generate a series of principles and policy documents to guide how respectful and inclusive Indigenous research should be conducted in Australasia. We will continue to endeavor to make this a priority moving forward. The next AVS meeting (AVS13) will be held in December 2026 in Adelaide, South Australia.
